# Improved detection of low-level *GNAQ* mosaicism in Sturge-Weber syndrome through affected tissue testing, deep sequencing, and HRM-qPCR

**DOI:** 10.1186/s13023-026-04530-z

**Published:** 2026-08-03

**Authors:** Stephanie Lilja, Sarah Glatter, Magdalena Vass, Birgit Pimpel, Bernhard Rosensteiner, Amina Paquay, Iris Schmidt, Robert Birnbacher, Reginald E. Bittner, Martha Feucht, Wolfgang M. Schmidt

**Affiliations:** 1 Neuromuscular Research and Diagnostics, Center for Anatomy and Cell Biology, Medical University of Vienna, Vienna, Austria; 2 Epilepsy Center. Member of ERN EpiCARE. Department Pediatric and Adolescent Medicine, Medical University of Vienna, Vienna, Austria; 3 Department of Paediatrics and Adolescent Medicine, Villach, Austria; 4 Center for Anatomy and Cell Biology, Medical University of Vienna, Währinger Straße 13, Vienna, 1090 Austria

**Keywords:** Sturge-Weber syndrome, *GNAQ* gene, Somatic mosaicism, High-throughput nucleotide sequencing, Nucleic acid melt analysis

## Abstract

**Background:**

Sturge-Weber syndrome (SWS) is a rare genetic neurocutaneous syndrome. While classical (type I) SWS is mainly caused by tissue-specific somatic mosaicism of a recurrent missense variant in the *GNAQ* gene, other genes have been implicated in SWS. Therefore, genetic testing at an early stage can support clinical management of affected children and genetic counselling of their families.

**Methods:**

Thirteen type I SWS patients were enrolled in this study. In the majority, blood-based genetic testing did not lead to genetic confirmation. DNA isolated from tissue samples (lesional skin biopsies in *n* = 12 samples or affected brain tissue in *n* = 1 sample) was subjected to next-generation sequencing at high coverage. We also established and validated a high-resolution melt analysis quantitative PCR (HRM-qPCR) assay for specific detection of the recurrent *GNAQ* variant.

**Results:**

In contrast to peripheral blood samples, tissue samples from all patients were found to harbor the hotspot *GNAQ* c.548G>A p.(Arg183Gln) variant at fractions ranging from 0.5% to 8.3%. Subsequently, presence of the variant was confirmed by HRM-qPCR, which was found to be sensitive enough to detect variants even at very low fractions (0.25%).

**Conclusion:**

Data from our case series emphasize the importance of tissue-based analysis by deep sequencing to detect somatic mosaicism in SWS and suggest that HRM-qPCR is a powerful technique for the targeted confirmation of low-level somatic variants.

**Supplementary Information:**

The online version contains supplementary material available at https://doi.org/10.1186/s13023-026-04530-z.

## Background

In contrast to Mendelian disorders caused by germline pathogenic variants that are present in all cells of an affected individual, somatic mosaic disorders result from postzygotic *de novo* variants arising during embryonic development (“somatic mosaicism”) [1, 2]. Depending on the stage of embryonic development in which a somatic variant arises, only a subgroup of tissues or cells are affected. Consequently, variants can be tissue-specific and in some situations might not be detectable in affected individuals, when analyzing DNA isolated from peripheral blood [1, 2].

Neurocutaneous disorders represent a prominent example of tissue-specificity for somatic mosaicism, where early embryonic genetic alteration leads to variants only present in brain and skin tissue [1–4]. Somatic variants in the KRAS-PI3K-AKT3-mTOR pathway were shown to be responsible for specific abnormalities in brain development and/or vascular malformations [5, 6]. Gain-of-function *GNAQ* variants can drive aberrant overgrowth in endothelial cells, resulting in dysfunctional blood vessel formations, which can manifest in the skin (visible as “port-wine birthmarks”), eyes and brain, causing ophthalmological and neurological deficits [7, 8].

Sturge-Weber syndrome (SWS), also known as cerebral trigeminal neuroangiomatosis, is a rare genetically determined neurocutaneous disorder [7, 8] that occurs in 1:20,000 to 1:50,000 live births. SWS equally affects males and females without population-specific predilection [9]. Clinical manifestation is divided according the Roach Scale Classification in different subtypes of SWS [10]. Nevertheless, up to 90% of SWS patients develop epilepsy within their first years of life. A single recurrent activating missense variant in *GNAQ*, NM_002072.5: c.548G>A p.(Arg183Gln), was found consistently as somatic variant at low levels (typically at ~ 3–9%, but often also even lower) in individuals with SWS [11–13]. Interestingly, variants in other genes like *GNA11* and *GNB2* have been detected recently in SWS patients, who differ in their clinical manifestations from classical type 1 SWS [10, 14, 15]. Considering this genotype-phenotype correlations in SWS, the diagnostic procedure should integrate the attempt to identify the causative genetic variant in addition to the exact description of the clinical phenotype [16].

Here, we report on a cohort of *n* = 13 individuals with SWS, in all of whom we confirmed somatic mosaicism for the c.548G>A p.(Arg183Gln) *GNAQ* variant either in skin biopsies (*n* = 12) or in surgically resected brain tissue (*n* = 1). Using a combination of deep next-generation sequencing (DNGS) at an average coverage of > 500-fold for selected target genes, followed by confirmation with high-resolution melt quantitative PCR (HRM-qPCR), we found that the *GNAQ* somatic variant can be present at very low levels (as low as 0.5%) in affected skin tissue, but was consistently not detectable in DNA isolated from peripheral blood samples (which was performed in *n* = 11). Due to the low variant allele fraction, conventional (“Sanger”) DNA sequencing was also not suitable for confirmation. We therefore established and validated a high-resolution melt (HRM) quantitative PCR (qPCR) protocol for reliably verifying the presence of the *GNAQ* hotspot variant with a detection limit of 0.25%.

## Methods

### Study population

Individuals clinically diagnosed with SWS according to the Roach Scale classification [10] were enrolled from the Department of Pediatrics and Adolescent Medicine, Medical University of Vienna, Austria and in one case from the Children’s Hospital, KABEG, LKH Villach, Villach, Austria. We investigated either skin (*n* = 12) or brain tissue resected during epilepsy surgery (*n* = 1). Written informed consent was obtained from all included participants or their legal guardians. All methods were carried out in accordance with relevant guidelines and regulations of the Medical University of Vienna, Austria and this study was approved by the ethics committee of the Medical University of Vienna (EK-Nr. 978/2009 EK-Nr. 1357/2021). This study has been performed in accordance with the Declaration of Helsinki.

### SWS classification

According to the Roach Scale classification, three different sub-types of SWS can be classified that vary widely with respect to clinical manifestations: Type 1 (the most common “classic” form of SWS), includes facial and leptomeningeal angiomas (LA) as well as the possibility of glaucoma or choroidal lesions. Normally, only one side of the brain is affected. Type 2 includes facial angioma (“port wine” birthmarks) with a possibility of developing glaucoma. There is no evidence of brain involvement. Symptoms include glaucoma, cerebral blood flow abnormalities and headache. Type 3 shows exclusively leptomeningeal angioma. The facial angioma is absent and glaucoma rarely occurs [10].

### DNA extraction from tissue samples and blood cells

Genomic DNA was extracted from lesional skin tissue biopsies (punch biopsy, 4–5 mm in diameter; facial skin from near the frontal hairline or from the posterior cheek near the auricle to minimize the risk of visible facial scarring), or in one case from affected brain tissue resected during surgery. The tissue samples were first incubated with a solution containing 97.5 µL “DirectPCR Lysis Reagent” (Viagen, Los Angeles, USA) 2.5 µL proteinase K (Qiagen, Hilden, Germany) and 100 µL sterile pyrogen-free distilled water (Ampuwa, Fresenius Kabi, Austria) for 1–2 h at 55 °C or overnight at 4 °C. After lysis, the sample was processed with the QIAamp DNA Mini Kit (Qiagen, Hilden, Germany) according to the manufacturer’s protocol (“cleanup-protocol”). DNA was eluted in 40 µL TlowE buffer (10 mM Tris-Cl pH 8.0, 0.1 mM EDTA). DNA from matched EDTA-blood samples (individuals P1-P4) was extracted using the Qiagen QIAamp DNA Mini protocol for blood samples. DNA concentrations were measured by the NanoDrop spectrophotometer.

### Exome sequencing and cancer gene panel next-generation sequencing

Exome sequencing (ES) was performed by construction of a DNA library using the Agilent SureSelect Clinical Research Exome Version 2 enrichment protocol (Agilent Technologies, Santa Clara, CA, USA) from blood in individuals P1-P4 and from skin in P5. Deep next generation sequencing (DNGS) was performed using a cancer-related gene panel containing 591 (Inview Oncoprofiling v4, Eurofins Genomics, Constance, Germany) in individuals P1-P4 and P6-P10 or 728 genes (Inview Oncoprofiling v5, Eurofins Genomics) in individuals P11-P13. Libraries were sequenced in 150 bp-paired end mode on an Illumina NovaSeq6000 sequencer (llumina, San Diego, CA, USA). Average on-target sequencing depth achieved was ~ 90-fold (ES) and ~ 1500-fold (DNGS), respectively. NGS libraries and raw data were generated by Eurofins Genomics Europe Sequencing (Constance, Germany).

Bioinformatic data analysis starting from *fastq* files was performed at the Neuromuscular Research and Diagnostics group (Medical University of Vienna) using an in-house bioinformatic pipeline for: (i) read mapping to the human reference genome (GRCh37/hg19) with BWA-MEM [17, 18], (ii) marking of duplicate reads and recalibration following the GATK best practice guideline [19], (iii) variant detection using the algorithms GATK HaplotypeCaller for ES or LoFreq (20), GATK Mutect2 [20], and VarDict [21] for DGNS [3], and (iv) annotation of variants using ANNOVAR [22] for comparison to reference databases (such as dbSNP, 1000 Genomes Project, gnomAD, and ClinVar). The Integrative Genomics Viewer (IGV) [23, 24] and LoFreq in pileup summary mode were used for visual inspection and supervised targeted analysis of the *GNAQ* hotspot variant (hg19_chr9:g.80412493C>T). For details on the algorithms used to detect variants, please refer to Supplementary Material.

### Sanger sequencing of the *GNAQ* hotspot variant

We designed region specific primers for confirming the presence of the somatic variant with bi-directional conventional DNA sequencing. PCR primers for amplification of a 150 bp PCR product were as follows: GNAQ_ex4_for: 5’-TAT CTT AAT GAC TTG GAC CGC GTA-3’, and GNAQ_ex4_rev: 5’-GTT TCA TGG ACT CAG TTA CTA CCT G-3’ Primers were synthesized by Merck (Darmstadt, Germany).

### High-Resolution Melt (HRM) analysis of qPCR for detection of the *GNAQ* hotspot variant

Because Sanger sequencing has its limitations for reliably detecting mosaic variants of less than ~ 10% [25], primers for amplifying a shorter amplicon (109 bp) were designed to ensure robust discrimination between reference and the hotspot variant in exon 4 of *GNAQ* in the HRM assay: GNAQ_HRM_for: 5’- TAT CTT AAT GAC TTG GAC CGC GTA-3’, GNAQ_HRM_rev: 5’- AGT CAA AGG GGT ATT CGA TGA TCC-3’. The qPCR and HRM were performed with a CFX Real-Time PCR system (Bio-Rad Laboratories, München, Germany) using Precision Melt Supermix (Bio-Rad Laboratories, München, Germany) under reaction conditions according to the manufacturer’s protocol, containing 2 pmol of each primer and 60 ng genomic DNA in 10 µL reaction volumes. Cycling conditions were 40 cycles at 10 s at 95 °C, 30 s at 60 °C and 30 s at 72 °C, after an initial denaturation at 95 °C 2 min. Melt curve analysis was performed at 0.2 °C increments at 10 s steps according to the manufacturer’s recommendations. Runs were setup in triplicates, with control DNA harboring the *GNAQ* variant, two reference DNA samples without the variant, and no template controls. Data were analyzed with the Precision Melt Analysis Software 1.3 (Bio-Rad Laboratories, Munich, Germany).

**Fig. 1:**
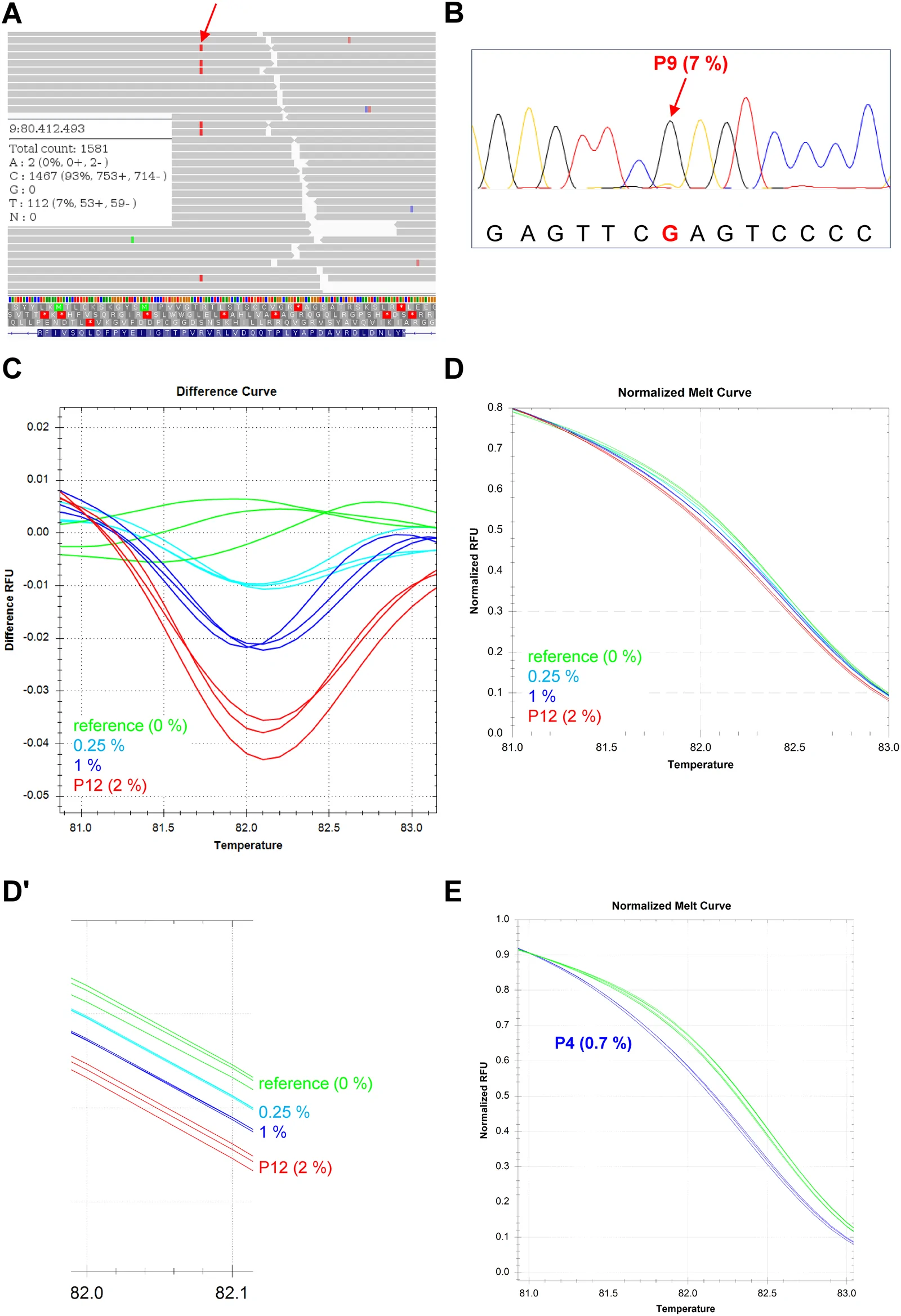
Detection of the *GNAQ* hotspot variant using HRM compared to DNGS and conventional DNA sequencing. (**A**) Detail of an IGV view showing part of *GNAQ* exon 4 harboring the hotspot variant (red arrow) after ultra-deep sequencing of DNA isolated from the skin biopsy of individual P9. The inset shows reference and variant depths at the respective position (hg19_chr9:g.80412493 C > T; c.548G > A p.(Arg183Gln), indicating a variant fraction of ~ 7%. (**B**) Trace view of conventional DNA sequencing of the same DNA sample after PCR-amplification. The arrow marks the position at which a small additional peak (for the variant A) in phase with the reference peak (a G) is visible. (**C**) Difference and normalized (**D**) melt curves after qPCR and HRM analysis of a positive sample (P12, variant fraction 2%, shown in red) mixed with normal reference DNA without the variant (0%, shown in green) at different ratios to simulate lower variant fractions. Melting curves differing from the normal reference (green) are visible for P12 (2%, red), as well as for the 1:2 (1%, shown in blue) and 1:8 dilutions (0.25%, shown in light blue). Curves for all triplicates are shown. The critical temperature segment between 82.0 and 82.1 °C of the normalized melting curve is enlarged in **D’**, showing that variant levels as low as 0.25% can be reliably detected by HRM. **E**. A representative HRM melting curve after qPCR of a DNA sample isolated from the skin biopsy of individual P4 (refer to sample 1 in Table 2) harboring the somatic *GNAQ* hotspot variant at low level (0.7%). RFU: relative fluorescence units

To determine the analytical sensitivity of the HRM assay, a serial dilution series was generated using DNA from a *GNAQ*-positive tissue sample mixed with normal reference DNA. Mixtures corresponding to expected variant allele fractions of 2%, 1%, 0.5%, 0.25%, 0.125%, and 0.0625% were analyzed. All runs were performed in triplicate. The resulting melting curves were compared to determine the lowest variant fraction that could be reliably distinguished from normal reference DNA samples. Results for variant fractions of 2%, 1% and 0.25% are shown in Fig. 1C and D. Further, consistent melting profiles were observed across repeated replicate measurements and between different runs, demonstrating both, high intra- and high inter-run reproducibility of the assay (see Supplementary Fig. 1).

## Results

### Clinical and genetic findings

Clinical findings are shown in Table 1. All patients included in this study, had vascular malformations affecting both face and brain, and thus were classified as type 1 SWS [10]. Two individuals had bilateral leptomeningeal angiomas. All patients developed epilepsy within their first years of life and most of them showed intellectual disabilities [11–13]. Magnetic Resonance Imaging (MRI) revealed calcifications in different parts of the brain in nine patients. All patients received antiseizure medication (ASM) and low-dose aspirin.

**Table 1:** Study cohort: demographic data and clinical findings

Individual		P1	P2	P3	P4	P5	P6	P7	P8	P9	P10	P11	P12	P13
Sex		M	M	F	F	F	F	M	F	M	M	F	F	F
Age at genetic testing	**years**	5.0	3.3	1.2	8.0	1.5	9.1	7.0	16.6	29.7	10.6	0.01	2.7	1.2
Genetic result	***GNAQ*** **NM_002072.5:** **c.548>A p.(Arg183Gln)**	2.25%	2.3%	8.3%	0.60%	3.9%	1.3%	2.3%	2.3%	7.0%	1.3%	0.65%	2.0%	4.5%
PWS		yes	yes	yes	yes	yes	yes	yes	yes	yes	yes	yes	yes	yes
Glaucoma		yes	no	no	no	yes	no	yes	no	yes	no	no	yes	no
MRI results	**leptomeningeal angiomas (LA)**	yes	yes	yes	yes	yes	yes	yes	yes	yes	yes	yes	yes	yes
	**LA hemisphere**	left	bilateral left> right	left	left	bilateral right> left	left	right	right	right	right	right	right	left
	**Calcifications**	none	none	yes (subcortical)	yes (cortical + subcortical)	yes	none	yes (cortical)	yes (falx cerebri, cortical)	yes	yes	yes (subcortical)	yes	yes
Seizures	**seizure type**	FS	FS, GTCS	FS	FS	FS, GTCS, epileptic spasms	FS, GTCS	FS	FS, GTCS	FE, GTCS	FS, epileptic spasms	FS	FS	FS, GTCS
	**SE**	no	yes	yes	no	yes	yes	no	yes	yes	yes	yes	yes	yes
	**Onset**	5 months	3 months	5 months	3 months	3 weeks	16 months	9 months	7 months	2 years and 2 months	4 months	2 weeks	2 years	8 months
ID, ASD		no	ID	ID, ASD	no	ID	no	no	no	ID	ID	ID	ID	no
Brain surgery		no/no paresis, language dominance?	posterior CC = > complete CC	HE left	HE left	no surgery	dominant HE, no paresis	no surgery	no surgery	disconnection TO right	HE right	HE right	no surgery	no surgery
			VNS				VNS			disconnection TPO right				
medication	**ASM history**	LEV, OCB, LCM	LEV, VGB, TPM, Steroids, Nitrazepam, KD	LEV, CBZ, VPA	CBZ, OCB	LEV, PB, OCB	LEV, VPA, CLB; PER	LEV	PB, LCM, Steroids, PER	LCM	VPA, BZD, TPM, KD	LEV, VGB, PB, KD	LEV, OCB	LEV, VitB6
	**Thrombo-ASS**	yes	yes	yes	no	yes	yes	yes	yes	yes	no	yes	yes	yes
	**mTOR inhibitor**	no	Everolimus	no	no	Everolimus	no	no	Everolimus	no	no	no	no	no

Abbreviations: M: male; F: female; PWS: port-wine stain; LA: leptomeningeal angiomas; ASM: anti-seizure medication; ASD: autism spectrum disorder; ID: intellectual disability

FS: febrile seizures; GTCS: generalized tonic-clonic seizures; FE: focal epilepsy; SE: Status epilepticus; CC: Corpus Callosotomy, HE: functional hemispherectomy

TPO: temporo-parieto-occipital; TO: temporo-occipital; LEV: Levetiracetam; OCB: Oxcarbazepine; LCM: Lacosamide; VGB: Vigabatrin; TPM: Topiramate; KD: ketogenic diet

CBZ: Carbamazepine; VPA: Valproic acid; PB: Phenobarbital; CLB; Clobazam; PER: Perampanel; BZD: Benzodiazepines; VitB6: vitamin B6, VNS: vagus nerve stimulation

Four patients (individuals P1-P4) initially had received a negative genetic result after exome sequencing (ES) of DNA extracted from peripheral blood (Table 2). Moreover, patients P5-P11 had received a negative ES result after blood-based testing conducted in other labs prior to this study (*not shown*). This prompted us to extend our diagnostic procedure to the analysis of affected tissue.

**Table 2:** Genetic findings

Individual	Sex	ES (blood)	DNGS						Confirmation	
		AD (ref/var)	tissue	AD (ref/var)	fraction	LoFreq	Mutect2	VarDict	Sanger	HRM
P1	M	202/0	skin (sample 1)	2050/46	2.2%	+	+	+	**-**	+
			skin (sample 2)	1976/47	2.3%	+	+	+	**-**	+
P2	M	156/0	skin	3371/81	2.3%	+	+	+	**-**	+
P3	F	172/0	brain	1796/163	8.3%	+	+	+	+	+
P4	F	173/0	skin (sample 1)	2025/14	0.7%	**-**	-/+	+	**-**	+
			skin (sample 2)	2360/12	0.5%	**-**	-/+	+	**-**	+
P5	F	(neg.)	skin	293/12*	3.9%	+	+	+	**-**	+
P6	F	(neg.)	skin	1837/25	1.3%	**-**	+	+	**-**	+
P7	M	(neg.)	skin	2127/51	2.3%	+	+	+	**-**	+
P8	F	(neg.)	skin	2027/47	2.3%	+	+	+	**-**	+
P9	M	(neg.)	skin	1364/103	7.0%	+	+	+	+	+
P10	M	(neg.)	skin	1861/25	1.3%	**-**	+	+	**-**	+
P11	F	(neg.)	skin	770/5	0.6%	**-**	-/+	+	**-**	+
P12	F	n.p.	skin	680/14	2.0%	+	+	+	**-**	+
P13	F	n.p.	skin	570/27	4.5%	+	+	+	**-**	+

Abbreviations: M: male; F: female; ES: exome sequencing; AD: allelic depth; ref: reference allele, var: variant allele (*GNAQ* hotspot variant); DNGS: deep next-generation sequencing; LoFreq, Mutect2, VarDict: variant calling algorithms used to detect the *GNAQ* hotspot variant in DNGS data (+: detected, -/+: detected only in force-calling mode, -: not detected); HRM: high-resolution melt analysis after qPCR

*: this sample was sequenced using a high-coverage exome sequencing (ES) protocol. (neg.): negative ES result after blood-based testing conducted in other labs prior to inclusion into this study (*not shown*)

### Identification of somatic variants in brain and skin tissue

Initially, we subjected DNA isolated from a lesional skin biopsy from individual P5 to ES. Due to the relatively high coverage (> 300-fold) at the position of the *GNAQ* hotspot variant achieved in this ES protocol, the pathogenic variant was detected (allelic fraction ~ 3.9%). However, we predicted that this ES approach, which often results in lower coverage at this specific position, might lead to false negative results in case of lower levels of the variant fraction. We therefore subjected all affected tissue samples to deep sequencing (minimum > 500-fold, but typically > 1500-fold) of a cancer gene panel that included *GNAQ* and *GNA11*, but not *GNB2* (Table 2). After visual inspection using the Integrative Genomics Viewer (IGV) and a straightforward analysis employing a pileup of the hotspot position, we found that the *GNAQ* missense variant c.548G>A p.(Arg183Gln) was detectable in all tissue samples tested, albeit at varying fractions, ranging from as low as 0.5% up to 8.3% (Table 2, and Fig. 1A). We tested several algorithms (*LoFreq*, *Mutect2*, *VarDict*) [20, 21, 26] for unsupervised variant detection and found that all performed well for variant levels above 2% but only *VarDict* can be configured for robust detection of very low levels (refer to individuals P4 and P11 in Table 2). The same in-depth data analysis procedure was performed for the described pathogenic variants in *GNA11* (p.Arg183Cys, p.Arg183Gln, p.Arg183Gly, or p.Gln209Arg), all of which were not detected in any of our SWS patients.

### Conventional DNA sequencing and HRM

All samples were then subjected to bi-directional DNA sequencing, which is routinely used as confirmatory method in our diagnostic workflow. We found that only variant levels > 7% can be reliably detected (Fig. 1B; Table 2), which is in line with reports that conventional sequencing does not represent a suitable diagnostic method in case of somatic mosaicism [25]. Therefore, we next established a high-resolution melt analysis quantitative PCR (HRM-qPCR) based assay for detection of the recurrent *GNAQ* missense variant c.548G>A p.(Arg183Gln), which proved to be able to confirm presence of the variant also at low fractions and successfully detected the variant even at levels as low as 0.25% (Fig. 1C and D). Thus, while most tissue samples in our series harbored somatic variant levels above ~ 2%, even for those below this level (see e.g. P4, Fig. 1E) HRM represents a relatively cost effective, rapid and sensitive method for detecting the *GNAQ* hotspot variant.

## Discussion

The detection of somatic pathogenic variants in affected skin tissue represents a key diagnostic criterion in neurocutaneous disorders such as SWS, with importance for diagnosis, management, and prognosis [27]. Thus, a genetic diagnosis can be helpful very early in the disease course. Since the first publication of somatic *GNAQ* variants in SWS in 2013, several follow-up papers showed that the hotspot variant c.548G>A p.(Arg183Gln) can be detected consistently, and a more recent review by Van trigt et al. summarized that about 80% of SWS patients harbor this somatic variant [8], which was corroborated recently [28]. Thus, our study contributes to this consistent series of findings, as we were able to detect the variant in all 13 SWS patients studied (100%). During our study, we observed a change in routine workup, as some SWS patients investigated herein, initially had received negative genetic results after blood testing. We suggest therefore to avoid relying on blood sample based genetic testing in SWS and to consider analyzing biopsies from affected skin. The level of detectable mosaicism depends on the sampled tissue and may be absent in unaffected tissue. Furthermore, variant allele fractions can vary between affected tissue samples, reflecting the mosaic distribution of the pathogenic somatic variant [29]. Thus, mosaicism may be unevenly distributed within the same affected tissue area, and current evidence does not support a clear correlation between variant allele fraction and disease severity. Although variant allele fractions varied across patients, these differences remain unclear and genotype-phenotype correlations are of high interest. Further studies are required to determine whether variant burden correlates with disease severity, prognosis, or therapeutic response. Accurate detection of low-level somatic mosaicism in affected tissues will be essential for these future studies and the development of precision medicine approaches in Sturge-Weber syndrome.

Because levels of mosaicism below ∼10% are difficult to reliably detect using conventional DNA (“Sanger”) sequencing [25, 30], we used HRM-qPCR for the confirmation of deep next generation sequencing results. This strategy allowed us to verify positive findings while minimizing the risk of false-positive calls caused by sequencing artefacts. HRM can discriminate between alleles due to the melting profile of the different nucleotides and is also suitable for the detection of somatic variants [31]. We established a HRM protocol to detect mosaicism of the *GNAQ* hotspot variant with high sensitivity. Analysis of the melting curve patterns allowed to reliably predict the presence of the variant in the amplified DNA fragments. In accordance with Joy et al. [32], who reported a detection limit of 0.3%, we identified a similar threshold of ~0.25% in detection of the *GNAQ* hotspot variant. Thus, HRM is an easily accessible, powerful and rather inexpensive tool to verify somatic variants, even at low levels of mosaicism.

Beyond the recurrent *GNAQ* c.548G>A p.(Arg183Gln) variant, the diagnostic workflow should also consider other activating somatic variants affecting the same signaling axis. Recently, a highly homologous gene encoding for different Ga subunit of G proteins *GNA11* was identified to harbor somatic variants in patients with SWS [9, 33, 34] and a genetic hotspot in *GNA11* p.(Arg183Cys) has been described to be related to an atypical SWS phenotype or SWS type 3 [35–37]. Atypical SWS was reported to be associated with *naevus roseus*, which darkens slowly while aging. Moreover, *cutis marmorata* and *naevus anaemicus* were observed in patients with *GNA11* variants [14, 35] and the patients showed milder courses and a delayed onset for neurological comorbidities [35]. Thus, although these variants appear to be rare, they are clinically relevant because they may be associated with atypical or milder SWS presentations. Therefore, a negative result for *GNAQ* c.548G>A should not automatically exclude molecular mosaicism [29, 38–40]. In our cohort, no pathogenic variants in the *GNA11* gene were detected. Fjaer et al. reported one patient carrying a *GNB2* variant with symptoms related to classical SWS, but this remains the only patient reported to date [15]. Due to lack of representation on the gene panels used in this study, we did not analyze *GNB2*.

Accordingly, we propose the following diagnostic approach: testing affected skin tissue rather than blood by using a targeted deep-sequencing panel including at least *GNAQ*, *GNA11* and *GNB2.* As skin biopsy is an invasive procedure and it may raise concerns regarding its acceptance, particularly in pediatric patients, but this study emphasizes that affected tissue-based molecular testing can significantly improve the detection of low-level somatic mosaicism.

## Conclusion

Patients with classical (type 1) SWS may present with progressive neurological and ophthalmological manifestations, including early-onset epilepsy, stroke-like episodes, headaches, cognitive impairment, hemiparesis, glaucoma, and visual field defects [35]. Although the recurrent *GNAQ* p.(Arg183Gln) variant is detected in the majority of patients, other pathogenic variants have been reported in individuals with overlapping but often milder phenotypes, further expanding the molecular spectrum of SWS-related disorders. Identifying the underlying genetic variant in SWS may provide valuable information for patients and clinicians regarding disease characterization and potential prognostic implications [16]. Although genotype-guided interventions are not currently part of routine clinical management, advances in molecular diagnostics may support the future development of targeted therapeutic approaches.

Collectively, findings from our study not only add to the knowledge of *GNAQ*-related SWS but also provide evidence that implementation of skin biopsy-based genetic testing is needed for genetic confirmation in SWS.

## Supplementary Information

Below is the link to the electronic supplementary material.

Supplementary Material 1: Supplementary Figure 1. High intra- and inter-assay reproducibility of the HRM *GNAQ* hotspot variant assay. **(A)** Normalized melt curves after qPCR and HRM analysis of a positive sample (P12, 2% shown in red) compared with a normal reference DNA (shown in green). All assays for both DNA samples were performed *n* = 7 times (with each sample measured in triplicates) in two independent runs carried out on two different days (#1, shown on the left and #2, right). **(B)** Box plots showing difference RFU values at the critical temperature of 82.2 °C (mean of triplicates) for all *n* = 7 assays for the reference DNA and P12 performed in runs #1 and #2. RFU: relative fluorescence units

Supplementary Material 2

## Data Availability

The *GNAQ* deep sequencing datasets generated and analyzed during the current study are available in the European Nucleotide Archive (ENA) repository, accession number: PRJEB85882 (https://www.ebi.ac.uk/ena/browser/view/PRJEB85882).

## References

[1] Poduri A, Evrony GD, Cai X, Walsh CA. Somatic mutation, genomic variation, and neurological disease. Sci (New York NY). 2013;341(6141):1237758. 10.1126/science.1237758 PMC3909954 23828942

[2] D’Gama AM, Poduri A. Brain somatic mosaicism in epilepsy: Bringing results back to the clinic. Neurobiol Dis. 2023;181:106104. 10.1016/j.nbd.2023.106104 36972791

[3] Freed D, Stevens EL, Pevsner J. Somatic mosaicism in the human genome. Genes (Basel). 2014;5(4):1064–94. 10.3390/genes5041064 PMC4276927 25513881

[4] Dou Y, Gold HD, Luquette LJ, Park PJ. Detecting Somatic Mutations in Normal Cells. Trends Genet. 2018;34(7):545–57. 10.1016/j.tig.2018.04.003 PMC6029698 29731376

[5] Jansen LA, Mirzaa GM, Ishak GE, O’Roak BJ, Hiatt JB, Roden WH, et al. PI3K/AKT pathway mutations cause a spectrum of brain malformations from megalencephaly to focal cortical dysplasia. Brain. 2015;138(Pt 6):1613–28. 10.1093/brain/awv045 PMC4614119 25722288

[6] Lim JS, Gopalappa R, Kim SH, Ramakrishna S, Lee M, Kim WI, et al. Somatic Mutations in TSC1 and TSC2 Cause Focal Cortical Dysplasia. Am J Hum Genet. 2017;100(3):454–72. 10.1016/j.ajhg.2017.01.030 PMC5339289 28215400

[7] Chen H, Sun B, Gao W, Qiu Y, Hua C, Lin X. Delineation of the phenotypes and genotypes of facial infiltrating lipomatosis associated with PIK3CA mutations. Orphanet J Rare Dis. 2023;18(1):189. 10.1186/s13023-023-02786-3 PMC10347770 37452404

[8] Van Trigt WK, Kelly KM, Hughes CCW. GNAQ mutations drive port wine birthmark-associated Sturge-Weber syndrome: A review of pathobiology, therapies, and current models. Front Hum Neurosci. 2022;16:1006027. 10.3389/fnhum.2022.1006027 PMC9670321 36405075

[9] Sanchez-Espino LF, Ivars M, Antonanzas J, Baselga E. Sturge-Weber Syndrome: A Review of Pathophysiology, Genetics, Clinical Features, and Current Management Approache. Appl Clin Genet. 2023;16:63–81. 10.2147/TACG.S363685 PMC10145477 37124240

[10] Taparia S, Agrawal S, Bista M. Sturge Weber Syndrome – Roach’s Type II Variant. Nepal J Dermatology Venereol Leprology. 2020;18(1):76–9.

[11] Duan Z, Xu K, Xie M, Tian X, Wang X, Feng J, et al. Clinical and pathologic features of Sturge-Weber syndrome in patients with refractory epilepsy. Am J Clin Pathol. 2024;161(5):469–82. 10.1093/ajcp/aqad174 38217527

[12] Nakashima M, Miyajima M, Sugano H, Iimura Y, Kato M, Tsurusaki Y, et al. The somatic GNAQ mutation c.548G > A (p.R183Q) is consistently found in Sturge-Weber syndrome. J Hum Genet. 2014;59(12):691–3. 10.1038/jhg.2014.95 25374402

[13] Shirley MD, Tang H, Gallione CJ, Baugher JD, Frelin LP, Cohen B, et al. Sturge-Weber syndrome and port-wine stains caused by somatic mutation in GNAQ. N Engl J Med. 2013;368(21):1971–9. 10.1056/NEJMoa1213507 PMC3749068 23656586

[14] Jordan M, Carmignac V, Sorlin A, Kuentz P, Albuisson J, Borradori L, et al. Reverse Phenotyping in Patients with Skin Capillary Malformations and Mosaic GNAQ or GNA11 Mutations Defines a Clinical Spectrum with Genotype-Phenotype Correlation. J Invest Dermatol. 2020;140(5):1106–e102. 10.1016/j.jid.2019.08.455 31726051

[15] Fjaer R, Marciniak K, Sundnes O, Hjorthaug H, Sheng Y, Hammarstrom C, et al. A novel somatic mutation in GNB2 provides new insights to the pathogenesis of Sturge-Weber syndrome. Hum Mol Genet. 2021;30(21):1919–31. 10.1093/hmg/ddab144 PMC8522634 34124757

[16] El Hachem M, Diociaiuti A, Galeotti A, Grussu F, Gusson E, Ferretti A, et al. Multidisciplinary, multicenter consensus for the care of patients affected with Sturge-Weber syndrome. Orphanet J Rare Dis. 2025;20(1):28. 10.1186/s13023-024-03527-w PMC11740666 39819452

[17] Li H, Durbin R. Fast and accurate short read alignment with Burrows-Wheeler transform. Bioinformatics. 2009;25(14):1754–60. 10.1093/bioinformatics/btp324 PMC2705234 19451168

[18] Li H. Aligning sequence reads, clone sequences and assembly contigs with BWA-MEM. 2013. https://arxiv.org/abs/1303.3997.

[19] Van der Auwera GA, Carneiro MO, Hartl C, Poplin R, Del Angel G, Levy-Moonshine A, et al. From FastQ data to high confidence variant calls: the Genome Analysis Toolkit best practices pipeline. Curr Protoc Bioinformatics. 2013;43(1110):11.0.1-.0.33. 10.1002/0471250953.bi1110s43 PMC4243306 25431634

[20] Cibulskis K, Lawrence MS, Carter SL, Sivachenko A, Jaffe D, Sougnez C, et al. Sensitive detection of somatic point mutations in impure and heterogeneous cancer samples. Nat Biotechnol. 2013;31(3):213–9. 10.1038/nbt.2514 PMC3833702 23396013

[21] Lai Z, Markovets A, Ahdesmaki M, Chapman B, Hofmann O, McEwen R, et al. VarDict: a novel and versatile variant caller for next-generation sequencing in cancer research. Nucleic Acids Res. 2016;44(11):e108. 10.1093/nar/gkw227 PMC4914105 27060149

[22] Wang K, Li M, Hakonarson H. ANNOVAR: functional annotation of genetic variants from high-throughput sequencing data. Nucleic Acids Res. 2010;38(16):e164. 10.1093/nar/gkq603 PMC2938201 20601685

[23] Robinson JT, Thorvaldsdóttir H, Wenger AM, Zehir A, Mesirov JP. Variant Review with the Integrative Genomics Viewer. Cancer Res. 2017;77(21):e31–4. 10.1158/0008-5472.CAN-17-0337 PMC5678989 29092934

[24] Robinson JT, Thorvaldsdóttir H, Winckler W, Guttman M, Lander ES, Getz G, et al. Integrative genomics viewer. Nat Biotechnol. 2011;29(1):24–6. 10.1038/nbt.1754 PMC3346182 21221095

[25] Rohlin A, Wernersson J, Engwall Y, Wiklund L, Björk J, Nordling M. Parallel sequencing used in detection of mosaic mutations: comparison with four diagnostic DNA screening techniques. Hum Mutat. 2009;30(6):1012–20. 10.1002/humu.20980 19347965

[26] Wilm A, Aw PP, Bertrand D, Yeo GH, Ong SH, Wong CH, et al. LoFreq: a sequence-quality aware, ultra-sensitive variant caller for uncovering cell-population heterogeneity from high-throughput sequencing datasets. Nucleic Acids Res. 2012;40(22):11189–201. 10.1093/nar/gks918 PMC3526318 23066108

[27] Kioutchoukova I, Foster D, Thakkar R, Ciesla C, Cabassa JS, Strouse J, et al. Neurocutaneous diseases: diagnosis, management, and treatment. J Clin Med. 2024;13(6). 10.3390/jcm13061648 PMC10971194 38541874

[28] Zhang B, He R, Wu R, Yang Z, Hu M, Zhang N, et al. Somatic GNA11/GNAQ variants in a cohort of Chinese children with phakomatosis pigmentovascularis. Pediatr Investig. 2024;8(2):117–25. 10.1002/ped4.12424 PMC11193378 38910853

[29] Galeffi F, Snellings DA, Wetzel-Strong SE, Kastelic N, Bullock J, Gallione CJ, et al. A novel somatic mutation in GNAQ in a capillary malformation provides insight into molecular pathogenesis. Angiogenesis. 2022;25(4):493–502. 10.1007/s10456-022-09841-w PMC9529792 35635655

[30] Cook CB, Armstrong L, Boerkoel CF, Clarke LA, du Souich C, Demos MK et al. Somatic mosaicism detected by genome-wide sequencing in 500 parent-child trios with suspected genetic disease: clinical and genetic counseling implications. Cold Spring Harb Mol Case Stud. 2021;7(6). 10.1101/mcs.a006125 PMC8751411 34697084

[31] Riahi A, Kharrat M, Lariani I, Chaabouni-Bouhamed H. High-resolution melting (HRM) assay for the detection of recurrent BRCA1/BRCA2 germline mutations in Tunisian breast/ovarian cancer families. Fam Cancer. 2014;13(4):603–9. 10.1007/s10689-014-9740-5 25069718

[32] Joy RA, Thelakkattusserry SK, Vikkath N, Bhaskaran R, Krishnan S, Vasudevan D, et al. Somatic mutation detection efficiency in EGFR: a comparison between high resolution melting analysis and Sanger sequencing. BMC Cancer. 2020;20(1):902. 10.1186/s12885-020-07411-1 PMC7510081 32962681

[33] Thorpe J, Frelin LP, McCann M, Pardo CA, Cohen BA, Comi AM, et al. Identification of a Mosaic Activating Mutation in GNA11 in Atypical Sturge-Weber Syndrome. J Invest Dermatol. 2021;141(3):685–8. 10.1016/j.jid.2020.03.978 PMC8483769 32771470

[34] Bolli A, Nriagu B, Britt AD, Toole AD, Treat J, Srinivasan A, et al. Mosaic pathogenic variants in AKT3 cause capillary malformation and undergrowth. Am J Med Genet Part A. 2023;191(5):1442–6. 10.1002/ajmg.a.63121 PMC10081949 36695285

[35] Dompmartin A, van der Vleuten CJM, Dekeuleneer V, Duprez T, Revencu N, Desir J, et al. GNA11-mutated Sturge-Weber syndrome has distinct neurological and dermatological features. Eur J Neurol. 2022;29(10):3061–70. 10.1111/ene.15452 35715928

[36] Langbroek GB, Stor MLE, Janssen V, de Haan A, Horbach SER, Graupera M, et al. Characterization of Patient-Derived GNAQ Mutated Endothelial Cells from Capillary Malformations. J Invest Dermatol. 2024;144(6):1378–e881. 10.1016/j.jid.2023.10.033 38013159

[37] Cousyn L, Leclercq D, Ta MC, Gilbert F, Di Meglio L, Marois C, et al. Late-Onset Status Epilepticus Associated With Isolated Leptomeningeal Angioma and Sturge-Weber Syndrome-Related GNA11 Pathogenic Variation. Neurology. 2023;101(22):1021–2. 10.1212/WNL.0000000000207839 PMC10727224 37813580

[38] Polubothu S, Al-Olabi L, Carmen Del Boente M, Chacko A, Eleftheriou G, Glover M, et al. GNA11 Mutation as a Cause of Sturge-Weber Syndrome: Expansion of the Phenotypic Spectrum of G(α/11) Mosaicism and the Associated Clinical Diagnoses. J Invest Dermatol. 2020;140(5):1110–3. 10.1016/j.jid.2019.10.019 PMC7187890 31838126

[39] Pilch J, Mizera J, Tota M, Donizy P. GNAQ/GNA11-related benign and malignant entities-a common histoembriologic origin or a tissue-dependent coincidence. Cancers (Basel). 2024;16(21). 10.3390/cancers16213672 PMC11544895 39518110

[40] Shah AD, Alexieff P, Tatachar P. Sturge-Weber syndrome: a narrative review of clinical presentation and updates on management. J Clin Med. 2025;14(7). 10.3390/jcm14072182 PMC11989965 40217631

