# Supplementary material for: Improved detection of low-level GNAQ mosaicism in Sturge-Weber syndrome through affected tissue testing, deep sequencing, and HRM-qPCR
Source: Orphanet J Rare Dis. 2026 Aug 3;21:397. doi: 10.1186/s13023-026-04530-z (PMC13733034; doi:10.1186/s13023-026-04530-z)
Supplement: Supplementary file 1 [file 13023_2026_4530_MOESM2_ESM.docx]

**BIONFORMATIC METHODS FOR VARIANT CALLING**

The following algorithms and settings were used for variant calling in the *GNAQ* gene:

(i) **LoFreq** (version 2.1.5, precompiled binary lofreq_star-2.1.5_linux-x86-64.tgz downloaded from [*https://github.com/CSB5/lofreq/tree/master/dist*](https://github.com/CSB5/lofreq/tree/master/dist)) with default parameters for base and mapping quality filters:

```
lofreq call \
  --ref "${ref_hg19_fasta}" \
  --region "9:80331003-80646374" \
  --out "${out_vcf}" \
  "${in_bam}";
```

**Pileups** for the *GNAQ* hotspot variant (**hg19_chr9:g.80412493C>T**) were generated as follows:

```
 lofreq call \
  --plp-summary-only \
  --ref "${ref_hg19_fasta}" \
  --region "9:80412483-80412503" \
  "${in_bam}" \
| grep 80412493 | cut -f5-9 - ;
```

Counts for C (reference) and T (variant) were summarized and are shown in the manuscript in **Table 2**, column AD (ref/var). As calculated from n=44 control datasets, we determined that the background for this pileup is of less than one variant read per 1000 reference reads.

(ii) **Mutect2** (version 4.6.2.0, download from [*https://github.com/broadinstitute/gatk/releases*](https://github.com/broadinstitute/gatk/releases)):

```
java -jar gatk-package-4.6.2.0-local.jar Mutect2 \
  --reference "${ref_hg19_fasta}" \
  --input "${in_bam}" \
  --intervals "9:80331003-80646374" \
  --alleles "${hotspots_vcf}" \
  --native-pair-hmm-threads $(nproc --all) \
  --output "${temp_vcf}";
```

followed by:

```
java -jar gatk-package-4.6.2.0-local.jar FilterMutectCalls \
  --reference "${ref_hg19_fasta}" \
  --variant "${temp_vcf}" \
  --output "${out_vcf}";
```

Note that we observed that the force-calling mode using the option "--alleles" in Mutect2 was necessary for variant detection in cases with very low variant allele fractions (with "${hotspots.vcf}" containing the *GNAQ* hotspot variant). All other parameters for calling and filtering were default. Java was version 21 (openjdk 21.0.4 2024-07-16 LTS).

(iii) Java port of **VarDict** (version: 1.8.3, downloaded from [*https://github.com/AstraZeneca-NGS/VarDictJava*](https://github.com/AstraZeneca-NGS/VarDictJava) and compiled according to the instructions on a PC running Ubuntu 22.04 LTS) with the threshold for allele frequency set to 0.4 %, the minimum number of variant reads set to 4, and all other settings set to default:

```
export LC_ALL=en_US.UTF-8;

VarDict \
  -G "${ref_hg19_fasta}" \
  -f 0.004 \
  -r 4 \
  -N "${sample_id}" \
  -b "${in_bam}" \
  -c 1 -S 2 -E 3 -g 4 \
  -th $(nproc --all) \
  -R "9:80331003-80646374" | \
teststrandbias.R | \
var2vcf_valid.pl \
  -N "${sample_id}" \
  -E \
  -f 0.004 \
> "${out_vcf}";
```
